# Epidemiologic analysis of central vein catheter infection in burn patients

**Published:** 2017-10

**Authors:** Maryam Roham, Mahnoush Momeni, Mohsen Saberi, Rahil Kheirkhah, Ali Jafarian, Hossein Rahbar

**Affiliations:** 1 Burn Research Center, Iran University of Medical Sciences, Motahari Burn Hospital, Tehran, Iran; 2 Rowan University Graduate School of Biomedical Sciences, New Jersey, USA

**Keywords:** Central Vein Catheter, Infection, Burn wound

## Abstract

**Background and Objectives:**

Currently, there are no well-defined guidelines or criteria for catheter-site care in burn patients, and there is little information about the epidemiology of central vein catheter (CVC) infection in such patients. This study aimed at addressing the epidemiological aspect of CVC infection in a sample of Iranian burn patients admitted to the largest referral burn center in Iran, Motahari Burn Center.

**Materials and Methods:**

A total of 191 burn patients were eligible for the study. Catheter related blood stream infection (CRBSI) was diagnosed according to suspected line infection, sepsis or blood culture growing bacteria, which could not have been associated with another site.

**Results:**

Of the 191 patients in this study, 45 males (23.68%) and 19 females (10%) had positive blood culture, confirming CV line infection. Patients who were burned by gas, gasoline ignition or burning Kerosene had the highest incidence of CV line infection. In contrast, patients burned by alcohol, pitch or thinner had the lower rate of CV line infection. Incidence of CV line infection was higher in patients with delay in presentation to the burn center (55.2%) when compared to those who presented without delay (22.8%). *Pseudomonas aeruginosa* was the most frequent colonizer of the wound culture (52.4%), the dominant strain of the first catheter tip culture (35%) and the dominant strain of the same day blood samples (53.8%). The mortality rate in patients diagnosed with CRBI was 21.9%.

**Conclusion:**

One of the important factors related to CV line infection is delay inpresentation to the burn center. The rate of CV line infection was 20.64 in catheter days.

## INTRODUCTION

There have been several reports addressing CVC infection leading to high mortality, morbidity and prolonged hospitalization (1, 2). Controlling and managing these infections has imposed a heavy financial burden on the government (3). In an effort to reduce the burden of these infections and improve the standard of care for patients, healthcare providers, insurers, regulators and patients, advocates have attempted to reduce the incidence of CRBSI (4). These attempts have spanned multiple disciplines in the healthcare field and include such efforts as appropriate placement and removal of CVC by the healthcare professionals, monitoring infection incidence and progress by infection control personnel, organization of infection control committees by healthcare managers and even patients, who assist physicians in caring for their inserted catheters (5, 6). The main goal of an effective preventive program is to eliminate CVC infection from all patient care areas. By providing an epidemiological analysis of CVC infection and educating health care personnel on the findings, we can move one step forward towards creating guidelines that help standardize the approach for providing care to at risk patients and ensure the existence of a minimum standard of care. The applications of this study are of particular importance in burn-related treatment centers due to the high risk of mortality and morbidity associated with CVC infection in these settings (7, 8). In other words, there is increased risk for multi-localized and extensive infections not only in inserted venous catheters, but also in other organs (9). Additionally, because venous catheters in burn cases are frequently inserted either just at or in proximity to the wound site, CVC infections are more common in burn patients (10).

Despite the prevalence of CVC infections and their high mortality, there are no well-defined guidelines or criteria for catheter site care and the frequency at which they should be changed in burn patients. Also, little information is available on the epidemiology of CRBSI in burn patients. Thus, the present study aimed at addressing the epidemiological aspects of CVC infection in a sample of burn patients treated at Motahari Burn Center in Iran.

## MATERIALS AND METHODS

This was a retrospective review of a prospectively collected database of the adult and pediatric burn patients admitted to the Motahari Burn Hospital in Tehran, who had a central vein catheter in one year. Of the 191 patients, 23 were younger than 12 years. The data collected included the demographic information, total body surface area (TBSA), percentage of the burn wound, location of catheter insertion, blood and wound culture on the day of catheter removal and the delay time between when patients were admitted to a hospital and when they were referred to the burn center. Data also included the time that the catheter was in place. If the CVL infection was suspected, the catheter had to be removed and replaced with another one. The reason for changing the catheter was also included in the collected data. Wound culture was performed using swab culture. CRBSI was considered positive in patients, whose condition raised suspicion for catheter infection or sepsis, or if the blood culture result was positive with no other source of infection (11).

For statistical analysis, quantitative variables were presented as a mean ± standard deviation (SD) and categorical variables were summarized by frequency. Continuous variables were compared using at test or Mann-Whitney U test whenever the data did not appear to have a normal distribution, or when the assumption of equal variances was violated across study groups. Categorical variables were compared using chi square test. Statistical analysis was performed using SPSS Version 16.0 (SPSS Inc., Chicago, IL). P-value less than or equal to 0.05 was considered statistically significant.

## RESULTS

**Fig. 1.**
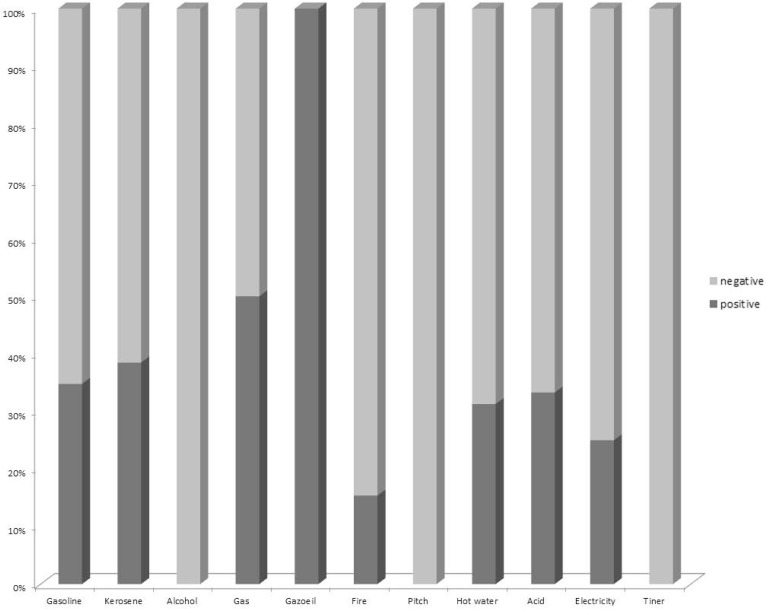
Results of central Vein Catheter infection according to burn categories

The most common type of burns were caused by an explosion (54 patients), followed by burns caused by a fire (52 patients), gasoline (23 patients) and hot water (16 patients), as demonstrated in Fig. 1. A significant difference was observed in the incidence of positive culture across different types of burning. The incidence of CVC infections was higher in patients whose burns were caused by gas, gasoline ignition and burning Kerosene, while the incidence-was lowest in patients with burns caused by alcohol, pitch, or thinner (p = 0.008).

Delay in presentation of the patient to the burn unit resulted in a higher rate of positive culture, with positive cultures observed in 55.2% of the patients with delay and 22.8% of patients without delay (p < 0.001). No significant difference was observed in the mean age difference of patients with or without CVC infection (30.56 ± 16.17 years vs.34.44 ± 15.13 years, p = 0.104). Of the 191 patients included in the study, 25 passed away (13.1%). Moreover, of the patients, who had CV line infection, 21.9% passed away. The rate of CVC infection in femoral catheters was 32.93% (Table 1), and the rate of CVC infection in internal jugular and subclavian sites combined was slightly higher at 29.13% (p = 0.048).

**Table 1.** Pathogens in femoral, internal jugular and subclavian catheter

**Source**	**First stage**	**%**	**Second stage**	**%**	**Third stage**	**%**
Blood sample						
	*Pseudomonas*	53.8	*Pseudomonas*	100	*Klebsiella*	100
	*Klebsiella*	23.1				
	*S. aureus*	7.7				
Catheter tip						
	*Acinetobacter*	35.9	*Pseudomonas*	50.0	*Pseudomonas*	50
	*Pseudomonas*	35.9	*Acinetobacter*	30.0	*Klebsiella*	50
	*S. aureus*	20.3	*Klebsiella*	10.0		
Burn wound						
	*Pseudomonas*	52.4	*Pseudomonas*	70	*Pseudomonas*	50
	*Acinetobacter*	38.1	*Acinetobacter*	30	*Klebsiella*	50
	*S. aureus*	4.8				

The dominant organism in the first catheter samples included *Acinetobacter baumannii* (35.9%), *P. aeruginosa* (35.9%) and *Staphylococcus aureus* (20.3%). The dominant organism in the blood samples included *P. aeruginosa* (53.8%), followed by *Klebsiella* (23.1%), and *S. aureus* (7.7%). The second blood culture, done at the same time of the second CVC catheter tip, revealed *P. aeruginosa* every time (100%). Cultures from the second CV line catheter tip showed *P. aeruginosa* (50.0%), *A. baumannii* (10%) and *Klebsiella*. Cultures from the third CV line catheter tip showed *P. aeruginosa* (50.0%) and *Klebsiella* (50.0%) (Table 2).

**Table 2.** Common pathogens isolated from blood samples, catheter tips and burn wounds

**CV Line Position**	*Acinetobacter baumannii*	*Pseudomonas aeruginosa*	*Staphylococcus aureus*	*Klebsiella*	*Entroccocus*
Femoral	20 (37%)	19 (35.1%)	11 (20.6%)	3 (5.5%)	1 (1.8)
Internal Jugular	3 (50%)	2 (33.4%)	1 (16.6%)		
Subclavian		2 (66.6%)	1 (33.4%)		

In total, 37.2% of the catheters were inserted at the burned site. A significant difference was found between positive CVC culture result and insertion of CVC in the burned area (p < 0.0001).

The first wound culture was done at the site nearest to the CV line insertion site and the results showed *P. aeruginosa* (52.4%), *A. baumannii* (38.1%) and *S. auerues* (4.8%). The second wound culture was done with the same criteria and the results showed *P. aeruginosa* (70%) and *A. baumannii* (30%).

There was a significant difference between TBSA and positive culture results (p= 0.001). Mean TBSA with positive culture results was 43.8%, while the mean TBSA in negative culture results was 38.7% (p = 0.001). In patients with positive tip culture, the mean and median were 17.47 days and 15.5 days, respectively. In the first culture of CVC, the positive culture was 20.64 per 1000 catheter days; in the second culture of CVC, the positive culture was 46 per 1000 catheter days; and in the third CVC culture, positive result was 80 in 1000 catheter days.

No significant difference was found in the mean age of the patients with positive and negative culture results from the first catheter tip (30.22 ± 16.05 years vs.29.20 ± 15.32 years, p = 0.788). Additionally, no difference was found in extensiveness of the burn, indicated by (TBSA) of patients with and without positive culture from the first catheter tip (43.85 ± 13.70 vs.46.04 ± 19.54, p = 0.556). Also, no difference was found in the mean age (22.00 ± 15.07 years vs.18.00 ± 13.08 years, p = 0.688) and mean TBSA (43.33 ± 11.90 vs.37.67 ± 10.79, p = 0.484) with regard to positive culture from catheter tip at the second stage. In total, no association was found among positive vascular catheter culture and the baseline variables of gender, the presence of inhalation injury, and cause of burn in both first and second stages

## DISCUSSION

Positive CRBSI was diagnosed whenever there was a suspicion of catheter infection, sepsis, or positive blood culture results with no other source of infection. The incidence of CRBSI per catheter day in burn intensive care unit (BICU) is much higher than its incidence in general ICU. This indicates a need for a separate set of guidelines for changing CV lines in burn patients. This study found that the rate of CRBSI was 20.6 per 1000 catheter days, compared to 15.4 per 1000 catheter days in O’Mara et al. study (11).

One option for changing a catheter is to rewire it. In rewiring a catheter, the line is left in place and only the CV catheter is replaced. There is conflicting findings on whether rewiring is superior to changing the whole catheter. In the Sheridan et al. study, it was supposed that changing CVP catheter with rewiring or changing catheter position was better for pediatric burn patients (12, 13). However, in a different study, it was found that catheter infection rates were 25.2 per 1000 catheter days in rewiring techniques, compared to 16.6 per 1000 catheter days in changing the whole catheter (11). In contrast, Eyer et al. indicated that there is a need for routine exchange of central line catheter and rewiring and that it does not reduce patients’ risk of infection (14). Rewiring techniques are not utilized frequently in our burn center, therefore, we could not collect data regarding rewiring of catheters.

In a multicenter study by Austin et al. in the United States, it was found that peripherally inserted central catheters were discontinued in 4.3% of burn patients due to central line associated bloodstream infection (15). In another study by Friedman et al., it was found that in patients with a TBSA > 60%, the incidence of CVC infection was 11.2 per 1000 catheter days, which was significantly higher than patients with a TBSA ≤ 60% (16). This study also revealed that the central venous catheters placed through burned skin instead of intact skin were 4 times more likely to be associated with CVC infection and that the most common infectious organism was *Acinetobacter*. This was similar to the finding of our study, as we found a significant difference between positive CVC culture results and insertion of CVC in burned areas of the skin. One possible explanation for the higher incidence of CVC infection in our burn patients, compared to general ICU patients, could be that the catheters were placed in burned areas of the skin or near it.

In a study by Tymonová et al. only 3.5% of patients had endogenous catheter colonization with positive peripheral blood culture and bacteremia; and the most frequent infecting pathogen in catheter tips was coagulase-negative Staphylococci (17). King et al. founda significant relationship between timing of central venous catheter exchange and frequency of bacteremia in burn patients (18). They found that the rate of catheter infection was 11% on the third day and 28% on the fourth day post insertion. They also found that CRBI occurred in 4% of the patients on the third day and 12% of the patients on the fourth day post insertion.

By comparing our results to those of previous surveys, 2 major points should be highlighted. Firstly, it seems that the rate of CVC infection is notably higher in Motahari Burn Center, when compared to developed countries. This indicates that the current approach is not successful in controlling CVC infections, presenting a need for a standardized set of guidelines to approach the care of catheter lines in burn patients. Secondly, it seems that the dominant pathogens that cause CVC infection are globally similar, and the most common pathogens include *Acinetobacter* and *S. aureus*. This can be attributed to the high global resistance of these pathogens to common antibiotics.

In our study, we found significant differences in the rate of infection based on location of the catheter in femoral, subclavian and jugular site, as demonstrated in Table 2, which is similar to findings of Greenhalgh and Deshpande and Goets articles (11, 19, 20). Previous studies also suggest that the risk of infection is lower in internal jugular catheters (21, 22). In the Greenhalgh study, it was found that the infection rate in adults is higher than the rate in children, possibly because of catheter insertion near the burned areas of the skin (23, 24) and because the adult patients had a higher TBSA and access via femoral catheters (25). In view of the positive tips on the second and third catheter culture, changing the protocol of central vein catheters in BICU should be seriously considered.

In this study, it was found that *P. aeruginosa* was the most common pathogen in CVC cultures taken the first time, followed by *A. baumnnii* and *S. aureus*. In the second CVC culture, when the site of the CVC was changed, the most common pathogen was *P. aeruginosa*, followed by *A. baumanii* and *Enterococcus*. In the third CVC culture, *P. aeruginosa* and *Klebsiella* were the most common pathogens. The wound culture taken from the wound nearest to the CVC insertion site revealed that the most common microorganisms were *P. aeruginosa*, *A. baumannii*, and *S. aureus*, which is identical to the first CVC cultures. This indicates that better wound infection control in burn patients can probably reduce the rate of positive CVC cultures. Additionally, the catheter insertion site should be as far from the burn wound as possible.

There are no current guidelines to determine when a patient catheter should be changed, and currently the catheters are changed only if there is suspicion of an infection. However, on average, infection is seen on catheter insertion on Day 17. We propose that to minimize the risk of infection, guidelines for catheter infection control must include a timeline to specify when catheters should be replaced.

There were some limitations in this study and this include: This retrospective study was conducted on new culture, first, second and third CVC culture in a burn center. It would be better to complete this study in a multicenter study to reduce all the essential factors. In Motahari Burn Center, rewiring is not performed as much, therefore, not enough data could be gathered about culture of the required CVL, and this should be included in further studies. Considering the higher rate of CRBSI in our center, changing the protocol for insertion, maintenance, and exchange is essential and the lines should be removed faster.
